# Towards optimal use of phosphorus fertiliser

**DOI:** 10.1038/s41598-020-74736-z

**Published:** 2020-10-20

**Authors:** Mart B. H. Ros, Gerwin F. Koopmans, Kees Jan van Groenigen, Diego Abalos, Oene Oenema, Hannah M. J. Vos, Jan Willem van Groenigen

**Affiliations:** 1 Soil Chemistry and Chemical Soil Quality Group, Wageningen University & Research, Wageningen, The Netherlands; 2 Soil Biology Group, Wageningen University & Research, Wageningen, The Netherlands; 3 Department of Geography, College of Life and Environmental Sciences, University of Exeter, Exeter, UK; 4 Department of Agroecology, Aarhus University, Aarhus, Denmark; 5 Wageningen Environmental Research, Wageningen University & Research, Wageningen, The Netherlands

**Keywords:** Element cycles, Agroecology

## Abstract

Because phosphorus (P) is one of the most limiting nutrients in agricultural systems, P fertilisation is essential to feed the world. However, declining P reserves demand far more effective use of this crucial resource. Here, we use meta-analysis to synthesize yield responses to P fertilisation in grasslands, the most common type of agricultural land, to identify under which conditions P fertilisation is most effective. Yield responses to P fertilisation were 40–100% higher in (a) tropical vs temperate regions; (b) grass/legume mixtures vs grass monocultures; and (c) soil pH of 5–6 vs other pHs. The agronomic efficiency of P fertilisation decreased for greater P application rates. Moreover, soils with low P availability reacted disproportionately strong to fertilisation. Hence, low fertiliser application rates to P-deficient soils result in stronger absolute yield benefits than high rates applied to soils with a higher P status. Overall, our results suggest that optimising P fertiliser use is key to sustainable intensification of agricultural systems.

## Introduction

Global food demand will rise substantially over the coming decades. Meeting this demand while decreasing the environmental footprint of agriculture is one of largest challenges of the twenty-first century^1–3^. A growing world population and changing diets are projected to double^4^ meat and dairy consumption between 2000 and 2050. As one of the main feed sources for livestock, grasslands play a key role in meeting this demand. With over 33 million km^2^, permanent grasslands account for ~ 25% of the world’s land cover. Over two thirds of this area is utilised for agriculture, making it the most dominant land use^5^. Sustainably increasing grassland productivity is therefore crucial to ensure future global food security^6,7^.

Phosphorus (P) is an essential nutrient, often limiting plant growth^8^. P fertilisation is therefore needed to sustain productivity in agricultural systems across the world. Because the world’s P reserves are decreasing, the importance of judicious P use will increase over the coming century. Although estimates of global P reserves vary, the costs of high quality P fertilisers will increase, as will the global demand for these fertilisers^9–12^. Differences in climate, geography, agricultural development, and fertilisation practices have led to great global imbalances of P in agricultural land^13–16^. In parts of Europe, North America, and China, historical applications of manure and fertilisers have resulted in positive P balances and increased risk of eutrophication of surface waters^17^. In many other regions, predominantly in tropical areas, farmers struggle to maintain soil P availability to sustain optimal rates of crop production^18^. Recent predictions suggest that global P inputs in grasslands will have to increase fourfold to support an 80% increase in grass yield projected for 2050^15^, which implies an urgent need to increase use efficiency of P fertiliser sources.

The large diversity in agronomic P status of soils across the world and the projected increase in cost and demand of P fertilisers necessitate a rethink of the use of P resources: are we applying fertilisers at the right rates to the right soils? The success of fertiliser application depends on conditions created by climate and management^19,20^ and is strongly governed by soil properties such as pH and concentrations of metal oxides and Ca in soil that can impact P availability to plants^8,21,22^. However, data for these relationships are fragmentary and country- or region-specific, and global assessments are lacking^23,24^. Here we use a meta-analysis on a global database of 67 studies and 1227 observations with a wide range of soil properties and climatic conditions to assess the general effect of P fertilisation on grassland production across the world. Furthermore, we identify soil-related driving factors that determine the success of fertiliser applications.

Our dataset included data from field grasslands all over the world (Supplementary Fig. 1). Most studies originated from Europe and North America, but due to several studies with many observations from the Australian continent, there were almost as many observations from Oceania. We analysed our dataset using two different metrics: the *response ratio* (RR) as measure for the relative increase in dry matter yield as a result of P fertilisation, and *P agronomic efficiency* (PAE) expressing the absolute yield increase per unit of P applied.

### Factors controlling the success of phosphorus fertilisation

**Figure 1:**
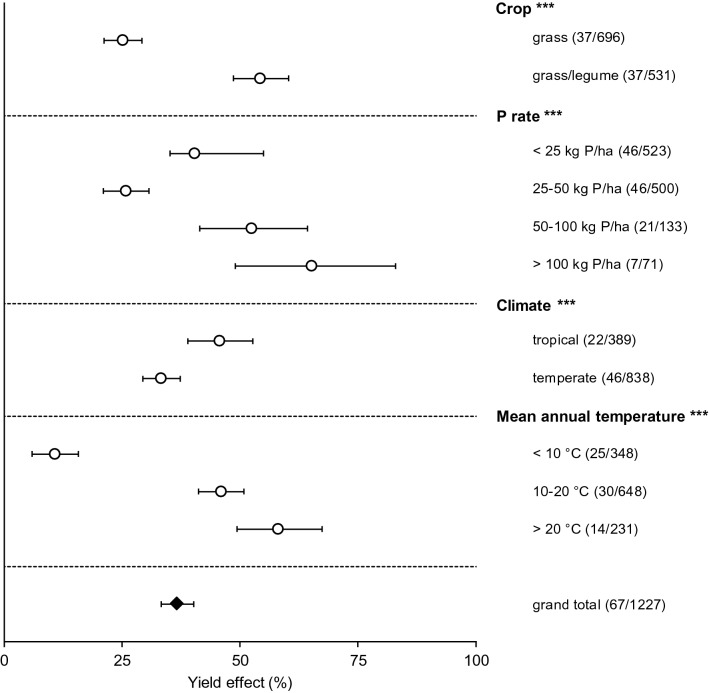
Impact of phosphorus (P) fertilisation for the controlling factors crop, P rate, climate, and mean annual temperature expressed as relative yield increase per category. The 95% confidence intervals are represented by the error bars, and the number of studies and observations per category are between parentheses; *,**,***Significant controlling factor effect at an α of 0.05, 0.01 and 0.001, respectively.

**Figure 2:**
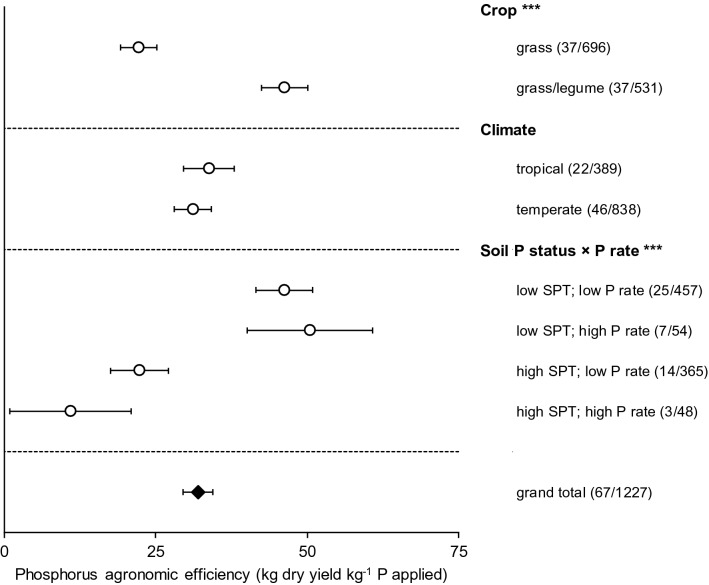
The Phosphorus agronomic efficiency (PAE) for different controlling factors per subgroup. The effect is expressed for crop, climate, and P status (Olsen-equivalent) × P rate (c). Low SPT: ≤ 10 mg P kg^−1^; high SPT: > 10 mg P kg^−1^; low rate: P rate ≤ 50 kg P ha^−1^; high rate: P rate > 50 kg P ha^−1^ The 95% confidence intervals are represented by the error bars, and the number of studies and observations per category are between parentheses; *, **,***Significant controlling factor effect at an α of 0.05, 0.01 and 0.001, respectively.

P fertilisation increased grassland yield by 37% (95% confidence interval: 33 to 40%; Fig. 1; Supplementary Table 3) averaged over all grasslands, soil types, and fertility levels, resulting in a PAE of 32 kg kg^−1^ (Fig. 2; Supplementary Table 4). In other words, dry matter yields increased by 32 kg per kg of P applied on average. Yield responses to P additions increased with P application rates: rates below 25 kg P ha^−1^ increased yields by 40% on average, whereas applying over 100 kg P ha^−1^ increased grass yield by 65%. An exception to this pattern were grasslands fertilised with 25–50 kg P ha^−1^, which responded to a lesser extent than those in other categories. This is likely an artefact due to a relatively high average soil P status of studies included in this category (Supplementary Fig. 3), which may have led to high yields in the control treatments. The PAE, on the other hand, decreased with P application rates (Supplementary Table 4): yields increased by 53 kg per kg P applied at rates lower than 25 kg P ha^−1^, but only by 12 kg kg^−1^ P at rates higher than 100 kg P ha^−1^. This indicates that finding a balance between P input and yield response is crucial for optimising fertiliser effectivity, as the agronomic efficiency decreases with higher application rates.

Systems that included legumes responded more strongly to P fertilisation than systems without legumes (Fig. 1). On average, P fertiliser increased yield in grass/legume systems by 54%, but only by 25% in grassland systems without legumes. These numbers corresponded with a PAE of 46 kg kg^−1^ for grass/legume and 22 kg kg^−1^ for grass-only systems, meaning that P fertilisation was roughly twice as effective in grasslands with legumes than in those without legumes. Legumes like alfalfa and clover are regularly included in grassland mixtures, mainly because they provide extra N inputs to the plant-soil system by establishing a symbiosis with N-fixing microorganisms^23^. These results likely reflect that legumes generally require more P than grasses, and can acquire it less easily due to thicker roots and shorter root hairs^11,25,26^.

In our database, more than half (36) of the studies included more than one N treatment. Overall, the N application rate had little effect on the response of grasslands to P fertilisation. There was no significant effect of N rate on the PAE (Supplementary Table 4). Yield responses to P fertiliser at N application rates over 200 kg N ha^−1^ were slightly but significantly smaller than at lower N rates (Supplementary Table 3). However, if N limitation of the grasslands would have played a prominent role, a general increase in response to P fertiliser with increasing N rate would have been observed. These results suggest that differences in yield responses were mainly driven by a response to P fertilisation rather than to N fertilisation.

### Geographical variation in responses

P application increased grassland yields in tropical regions (i.e. latitudes ≤ 35°) significantly more strongly than in temperate grasslands (Fig. 1, Supplementary Table 3). However, because yields of tropical grasslands were relatively low, the PAE of fertiliser application did not differ significantly between the two regions (34 and 31 kg kg^−1^ for tropical and temperate regions, respectively; Fig. 2, Supplementary Table 4). These results likely reflect that soils in (sub)tropical regions are often highly weathered, nutrient-poor, and have a low P availability due to high abundancy of adsorbents like Al and Fe oxides^8^. In contrast, decades of manure and fertiliser applications have resulted in a build-up of soil P levels well beyond crop requirements and a corresponding decrease in yield response to P fertiliser application^17,27^ in many temperate regions (e.g. North America, Europe, and New Zealand). The differences in response of temperate and tropical grasslands are also reflected in the results for mean annual temperature (MAT; Fig. 1, Supplementary Table 3), with grasslands in colder regions (MAT < 10 °C) showing the weakest relative response to P addition and grasslands with a MAT of > 20 °C reacting the strongest. Higher temperatures may lead to more rapid plant production and to an increase in mineralisation of organic matter. Correlation analysis of the controlling factors showed that MAT and latitude among our studies were strongly correlated (Supplementary Fig. 4; Spearman’s ρ = -0.95).

Yield responses to P fertilisation were significantly smaller in Asia, North America, and Europe (+ 15 to + 29%) than in South America, Oceania, and Africa (+ 58 to + 94%). The PAE ranged from 12 kg kg^−1^ for studies in Asia to 74 kg kg^−1^ for studies in Oceania and even 117 kg kg^−1^ for the one African study included in our dataset (Supplementary Table 4). The continents with grasslands that showed a strong response to P fertilisation roughly coincide with the areas that have relatively low P inputs and outputs, as modelled by Sattari et al.^15^. Taken together, these results imply that Africa and Oceania with low P inputs responded strongly to P fertilisation whereas grasslands in Europe, North America and Asia with relatively high P inputs over the past decades, showed a weak response to P fertilisation.

### Do we apply phosphorus fertilisers to the right soils?

We used various soil parameters as controlling factors (Table 1) to identify what soil properties drive differences in yield response to P fertilisation. One of the most important parameters is the agronomic P status of the soil, which is commonly determined with a soil P test (SPT). Because soil type, climate, and crop response vary considerably across the world, each country and sometimes even region has its own SPT method and classification system^28,29^. Given this large variety of SPT procedures (and resulting P concentrations) in use, we applied conversion formulas published in peer-reviewed papers to express reported SPT values in our database as ‘Olsen-equivalent’ P values wherever possible (see Supplementary Methods).

**Table 1:** Controlling factors and categories distinguished in the meta-analysis.

Controlling factors	Categories			
Crop	Grass	Grass/legume		
P application rate	≤ 25 kg P ha^−1^	25–50 kg P ha^−1^	50–100 kg P ha^−1^	> 100 kg P ha^−1^
Climate	tropical (≤ 35°)	temperate (> 35°)		
Mean annual temperature	< 10 °C	10–20 °C	> 20 °C	
P status (Olsen-equivalent)	≤ 5 mg P kg^−1^	5–10 mg P kg^−1^	10–25 mg P kg^−1^	> 25 mg P kg^−1^
P status × P rate^a^	low SPT, low P rate	low SPT, high P rate	high SPT, low P rate	high SPT, high P rate
Soil pH	≤ 5	5–6	6–7	> 7
Soil OM content	≤ 2%	2–5%	5–10%	> 10%
Soil clay content	≤ 10%	10–25%	> 25%	
N application rate	≤ 50 kg N ha^−1^	50–100 kg N ha^−1^	100–200 kg N ha^−1^	> 200 kg N ha^−1^

^a^SPT = Soil phosphorus test. Low SPT: ≤ 10 mg P kg^−1^; high SPT: > 10 mg P kg^−1^; low rate: P rate ≤ 50 kg P ha^−1^; high rate: P rate > 50 kg P ha^−1^.

**Figure 3:**
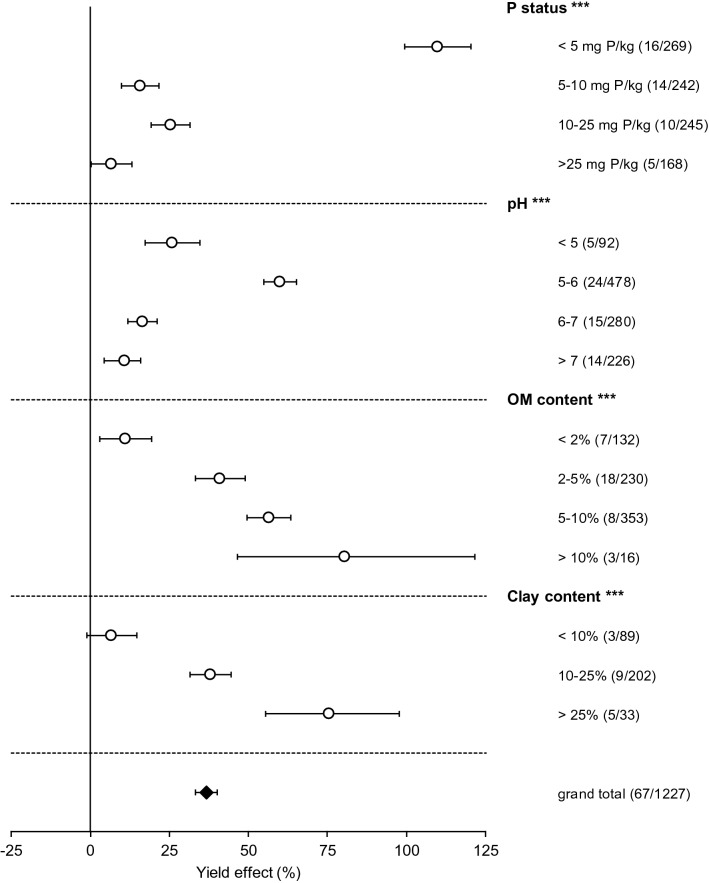
Effect of different soil characteristics on the impact of phosphorus (P) fertilisation. The effect is expressed for soil P status based on Olsen P-equivalent, soil pH, organic matter content, and clay content. The 95% confidence intervals are represented by the error bars, and the number of studies and observations per category are between parentheses; *, **,***Significant controlling factor effect at an α of 0.05, 0.01 and 0.001, respectively.

Grasslands on soils with low SPT values (≤ 5 mg P kg^−1^) responded strongest to P fertilisation with a yield increase of 110% on average (Fig. 3, Supplementary Table 3). Conversely, P additions to soils with SPT values > 5 mg P kg^−1^ increased yields by 7–25%. Although yield response decreased dramatically with increasing SPT values, the responses at relatively high SPT values (10–25 and > 25 mg P kg^−1^) were still statistically significant. Critical values (that is, SPT levels for which the yield is 95% of the maximum yield) for grass of 23–25 mg kg^−1^ Olsen P have been reported previously for English grasslands^30^, which coincides with the limited yield response for soils in the highest SPT category. A study of 25 Spanish soils also showed an average critical SPT of 24 mg Olsen P kg^−1^ for ryegrass, although there was a wide spread for individual soils, ranging from 11 to 46 mg kg^−1^^31^. For a range of Australian grassland species, however, lower critical SPT values (between 9 and 15 mg kg^−1^) have been determined^32^. This variety of critical SPT values found in literature illustrates that the effect of P fertilisation is strongly dependent on soil, climate, and even grassland species. Therefore, our results here do not give a hard SPT limit beyond which further P applications are rendered ineffective, but do indicate a strong decrease in effectiveness at higher SPT values.

The strong yield response to P fertilisation on soils with low SPT values was not merely the result of a low yield of the control treatments. PAE was also highest (75 kg kg^−1^) for soils with SPT ≤ 5 mg P kg^−1^ (Supplementary Table 4) and fertilisation on these soils was 3 to 8 times as effective as on soils with higher SPT values in terms of absolute yield increases. Without correcting for the P application rate, absolute yield responses (average yield of treated plots minus average yield of control plots) to P fertilisation varied substantially (− 2.7 to 11.3 tonnes ha^−1^; Supplementary Fig. 5). The largest response (on average 2.7 tonnes ha^−1^ increase) and variation to P fertilisation were found for soils in the lowest SPT category. The yield response decreased with higher SPT (Supplementary Fig. 5). Figure 2 shows that both relatively low (≤ 50 kg P ha^−1^) and relatively high (> 50 kg P ha^−1^) P application rates on soils with a low P status (≤ 10 mg P kg^−1^ Olsen-equivalent) were more effective than any P fertilisation rate on soils with a relatively high P status (> 10 mg P kg^−1^ Olsen-equivalent). The high PAE of large application rates on soils with a low P status (Fig. 2) may be the result of the binding behaviour of P in soil: in soils with a low P status (where relatively more P adsorption occurs), relatively high P inputs are required to raise the level of plant-available P, so grassland on these soils will benefit relatively more from high application rates. Conversely, applying large amounts of P to soils with a relatively high P status (> 10 mg P kg^−1^) showed a low PAE.

Yield responses to P applications were highest on grasslands with a soil pH of 5–6 (60% yield increase; Fig. 3) whereas lower and higher pH levels resulted in lower (11–26%) yield responses. We observed the same pattern for PAE, where studies with a pH of 5 to 6 had a 50 kg yield increase per kg of P fertilised, whereas for soils with a pH above 7 this was only 11 kg (Supplementary Table 4). Soil pH is a crucial parameter in determining the availability of P to crops^8^. In acidic mineral soils, binding of P to Fe and Al (hydr)oxides is often the main factor that governs the level of plant available P. In contrast, in soils with pH values above 7, P is more likely to form poorly soluble Ca-P precipitates, decreasing plant available P. The relative availability of soil P is highest at soil pH levels of 5 to 7^33,34^, which would imply that around this pH fertiliser P application would yield the strongest responses.

We found a positive correlation between the soil organic matter (OM) content and yield response to P fertilisation (Fig. 3). On average, P application increased yield by only 11% on soils with an OM content below 2% (PAE was 7.2 kg kg^−1^ on average and this effect was not statistically significant). Yield responses were much higher (41–80%) in soils with an OM content of > 5%. The PAE was 9 times as high in soils with > 5% OM as in soils with < 2% OM. The positive relationship between OM and response to P fertilisation likely reflects the competition between P and OM for binding sites on soil reactive surfaces. Naturally present OM can decrease the sorption of P and increase its solubility^35,36^. Consequently, P application to soils with relatively high OM contents is likely to increase levels of soil soluble P that is readily available to crops, causing P fertilisation to become more effective. Moreover, higher OM levels in soils can improve soil structure and water holding capacity, enhance aeration and infiltration, and mediate soil erosion^37,38^. Soils with a relatively high OM content may also have a higher nutrient mineralisation rate. All these effects may alleviate any constraints of plant growth besides P limitation, rendering P applications more effective.

Lastly, the yield response to P fertilisation increased with soil clay content. In fact, in soils with a clay content below 10%, grassland production was not significantly increased by P fertilisation, whereas in soils with more than 25% we found a yield response of 75% (Fig. 3; Supplementary Table 3). Clay helps to retain water and nutrients in soil, and thereby decreases the risks of drought and nutrient limitations. As these growth-limiting factors are reduced, P fertilisation may become more effective.

### Managing future phosphorus inputs in grasslands

Expansion and intensification of grassland systems over the coming decades is likely to occur due to the growing world population and an accompanying demand for meat and dairy products^15^. Transitioning towards increasingly sustainable ways to provide in this demand requires favourable socio-political constructs and appropriate rewards for producers. This transition may take on different forms in various parts of the world^6^ and there are many different pathways and advances that can contribute to increasing use efficiency of P in grassland systems, e.g., breeding or selection of species with favourable root traits, lowering crop P demands, proper re-use of P resources, or development of new P fertilisers^11,39,40^. In general, achieving high production while minimising P inputs, as well as losses to the environment, should be a unifying characteristic of this process.

Effective use of P in manure and fertilisers is essential in this transition. Our study provides broad and universal guidelines on where to apply P, and reveals that there is much to be gained from applying a limited resource more judiciously. Applying P to the soils with the lowest P status (Olsen-equivalent of ≤ 5 mg P kg^−1^) proves most effective in terms of absolute yield increases. Additionally, grasslands on soils with a relatively high OM content and a pH between 6 and 7, as well as grasslands that include legumes, represent the agricultural contexts where P fertilisation may achieve its highest potential.

Efficiently managing P resources in grasslands, for instance by keeping P surpluses (P applied – P removed through yield) small, increases the possibility of sustainable intensification. This means that fertilisation strategies should not let the soil P status increase to levels above the critical SPT values^30^. For soils with a P status around this critical value, application rates that replace the plant P offtake would be acceptable. Higher application rates for soils with a low P status will raise SPT levels over time, resulting in more optimal yields. However, in many intensively managed agricultural systems, application of P beyond the critical point occurs regularly as a result of high animal numbers per area and associated manure surpluses^27,41^. For these regions, regulating P inputs depending on SPT levels should be, and often is, a way to help reduce P losses.

A spatially detailed map of the soil P status of the world’s grasslands, which is currently lacking, would be an enormous step towards increasing the efficiency of P fertiliser use in the face of a booming population and dwindling reserves. The development of such maps and the improvement of its spatial accuracy are ongoing^42,43^ and will pave the way to ensure the sensible use of an increasingly scarce resource. Combining this with existing maps for OM content, pH, and clay would allow us to locate areas with the strongest responses to P fertilisation. As of now, it is difficult to get a more explicit global overview of where these areas are located. Attempts have been made to compare the agronomic P status of soils across or within continents^42^, but these attempts are often impeded by a lack of comparable and reliable data^42,44^. Alternatively, grassland P balances or budgets are often modelled or compared^13,15,16^, because these data are more easily obtainable. Still, these data often have poor coverage or spatial resolution and do not consider soil factors, which are of paramount importance for efficient P use as shown in our study.

## Methods

We searched the ISI Web of Science database for peer-reviewed publications reporting the effect of P fertilisation on the yield of grassland with 31 December 2016 as a cut-off date. The search was limited to field studies of at least one growing season that included a control treatment (experimental treatments differing only in P fertilisation) and reported the P fertilisation rates. This resulted in a database of 67 studies with 1227 pairwise comparisons (Supplementary Table 2). The number of observations obtained per study ranged from 1 to 204. For the selected studies, details on the experimental conditions were collected, which included information on location, presence of legumes, fertiliser form and rates, and soil characteristics such as SPT, OM, and pH. These data were used to define the controlling factors based on which we assessed the effect of P fertilisation on grassland production (Table 1). The magnitude of the total effect and the effect of the controlling factors was determined using two effect sizes. First, we used the natural logarithm of the response ratio (R)^45^:

$$\ln \left( R \right) = \ln \left( {\frac{{\overline{{x_{e} }} }}{{\overline{{x_{c} }} }}} \right)$$

with $\overline{{x_{e} }}$ and $\overline{{x_{c} }}$ being the mean yield of the experimental (P fertilised) and control treatments, respectively. Additionally, we used the P agronomic efficiency (PAE) to express the gain in yield per unit of P applied:

$$PAE = \frac{{\overline{{x_{e} }} - \overline{{x_{c} }} }}{P rate}$$

with $\overline{{x_{e} }}$ and $\overline{{x_{c} }}$ in kg ha^−1^ and P rate in kg P ha^−1^. These effect sizes were weighted by the inverse of their variance^45^. In addition, study was incorporated as an independent factor. A significant effect of P fertilisation was assumed when the 95% confidence interval did not overlap with zero. We considered differences between subgroups significant if the two 95% confidence intervals did not overlap. All analyses were done using the “metafor” package in R^46^. More information on the methods, SPT conversion formulas, as well as the limitations of our study can be found in the Supplementary Information.

## Supplementary information

Supplementary information.

## Data Availability

The dataset analysed during the current study is available from the corresponding author on reasonable request.
