# Supplementary material for: Towards optimal use of phosphorus fertiliser
Source: Sci Rep. 2020 Oct 20;10:17804. doi: 10.1038/s41598-020-74736-z (PMC7576788; doi:10.1038/s41598-020-74736-z)
Supplement: Supplementary file 1 — Supplementary information. [file 41598_2020_74736_MOESM1_ESM.docx]

**Supplementary Information to ‘Towards optimal use of phosphorus fertiliser’.**

**Title:** Towards optimal use of phosphorus fertiliser

**Authors:** Mart B.H. Ros^1,2^*; Gerwin F. Koopmans^1^; Kees Jan van Groenigen^3^; Diego Abalos^2,4^; Oene Oenema^2,5^; Hannah M.J. Vos^1,2^; Jan Willem van Groenigen^2^

^1^ Soil Chemistry and Chemical Soil Quality Group, Wageningen University & Research, Wageningen, Netherlands

^2^ Soil Biology Group, Wageningen University & Research, Wageningen, Netherlands

^3^ Department of Geography, College of Life and Environmental Sciences, University of Exeter, Exeter, United Kingdom

^4^ Department of Agroecology, Aarhus University, Aarhus, Denmark

^5^ Wageningen Environmental Research, Wageningen University & Research, Wageningen, Netherlands

*Corresponding author

Email: mart.ros@wur.nl

**Supplementary Methods**

**Data compilation.** We searched the ISI Web of Science database for published peer-reviewed papers with the topic matching the query ‘phosphorus AND grass* AND (fertili*er OR fertili*ation) AND (uptake OR yield)’, for all years, to a cut-off date of 31 December 2016. We selected publications from the query results that fitted all of the following criteria: (i) field studies with an experimental duration of at least one growing season; (ii) studies reporting yield of grass and/or grass/legume mixtures as response variable; and (iii) studies with experimental treatments for which a suitable control treatment could be identified. Control treatments were deemed suitable if experimental factors (i.e. site, sampling time, soil characteristics, and N application rate) were identical to those in the experimental treatment, and the control treatment did not receive any P fertilisation. This resulted in a database containing 67 studies and 1227 side-by-side comparisons of experimental and control treatments (Supplementary Table 2). From these studies, we collected the mean yield, the standard deviation (SD) or standard error (SE), and the number of replicates (n) for the experimental and control treatments. When SD or SE values were missing, proxy SD values were conservatively substituted using the mean of the observation and 150% of the averaged variance across the entire dataset. Figure error bars that were not labelled were assumed to represent SEs, rather than SDs ($SD=SE\times\sqrt{n}$).

**Controlling factors.** In addition to yield means and measures of variance, we extracted soil and environmental data that have been suggested to control the response of plant yield to P fertilisation. For all observations the parameters crop (i.e. grass or grass/legume mixture), P application rate (P rate), and latitude were collected. Data on average annual temperature at the study sites was extracted from the papers. In cases where no temperature data were provided, an approximate average temperature was extracted from an online database^1^. When available, we also included soil P test (SPT) data on soil P status (value and extraction procedure), soil pH (value and extraction method), soil OM content, soil clay content, and N application rate (N rate). All these parameters were used as controlling factors to make different categorical groups (Table 1) which we used to analyse the data. We gathered data on additional variables, such as application rates of K and CaCO_3_, grass species and seeding density, and soil Fe and Al content. However, these values were not reported consistently enough throughout the studies in the database to be included in the analysis.

The agronomic P status of a soil is commonly determined by a SPT. Based on the resulting SPT value, soil is classified as deficient or sufficient, depending on how the yield of a crop responds to P fertilisation at this SPT, which requires a series of P fertilisation trials^2,3^. Because soil type, climate, and crop response vary considerably across the world, each country and sometimes even region has its own SPT method and classification system^3,4^. The amount of P extracted by different SPTs varies strongly and depends on the nature of the extraction solution as well as the extraction procedure^2^. In the included studies, we encountered a wide array of SPT procedures applied across the world (Supplementary Figure 2). To be able to compare our response variables for grass yield (defined below) based on the soil P status, we transformed SPT values using the various extraction methods to an ‘Olsen P-equivalent’ with the help of conversion equations taken from peer-reviewed papers. Although the Olsen P test may produce unreliable results at low soil pH, we chose this test as a reference because it was the most frequently used extraction in our database, and it is used widely across the world. We selected the equations based on largest the number of samples included (n), the quality of fit of the equation (R^2^ > 0.7), and whether the SPT data to be converted fell within the range of SPT values used to derive the equation. Only direct conversions to Olsen P were used. The resulting selected equations are:

From Otabbong et al.^5^, for P-AL (mg kg^-1^) to P Olsen (mg kg^-1^):

$P_{OlsenEq}={(12.678+0.599\sqrt{P_{AL}}+0.232\sqrt{clay\%}-1.985\times{pH}_{H2O})}^{2}$ (1)

(n=82; R^2^=0.94)

From Wolf and Baker^6^, for Bray P1 (mg kg^-1^), Mehlich 1 (mg kg^-1^) and Mehlich 3 (mg kg^-1^) to Olsen P (mg kg^-1^):

$P_{OlsenEq}=0.302\times P_{BrayP1}+2.915$ (2)

(n=91; R^2^=0.72)

$P_{OlsenEq}=0.658\times P_{Mehlich1}-0.408$ (3)

(n=91; R^2^=0.87)

$P_{OlsenEq}=0.325\times P_{Mehlich3}+2.010$ (4)

(n=91; R^2^=0.79)

From Moody et al.^7^, for Colwell P (mg kg^-1^) to Olsen P (mg kg^-1^):

$P_{OlsenEq}=0.349\times P_{Colwell}+1.021$ (5)

(n=468; R^2^=0.71)

From Qian et al.^8^, for Modified Kelowna P (mg kg^-1^) to Olsen P (mg kg^-1^):

$P_{OlsenEq}=1.10\times P_{ModKelowna}-0.90$ (6)

(n=253; R^2^=0.92)

From Humphreys et al.^9^, for Morgan P (mg l^-1^) to Olsen P (mg l^-1^):

$P_{OlsenEq}=7.14\times P_{Morgan}-7.86$ (for values ≤ 10 mg l^-1^) (7)

(n=236; R^2^=0.76)

$P_{Morgan}=5.5-0.22\times P_{OlsenEq}+0.008\times{P_{OlsenEq}}^{2}$ (for values > 10 mg l^-1^) (8)

(n=368; R^2^=0.94)

More details about the SPT conversion can be found in Supplementary Table 1. For some studies it was necessary to convert soil P status from mg l^-1^ to mg kg^-1^ using the soil:solution ratio (SSR) employed during the extraction procedure, or from kg ha^-1^ to mg kg^-1^, using bulk density and sampling depth. If bulk density values (g cm^-3^) were unavailable, a value was calculated using an estimated mineral bulk density based on soil texture in combination with an assumed organic matter (OM) density (0.224 g cm^-3^)^10^:

$Soil bulk density=\frac{100}{\frac{\%OM}{OM bulk density}+\frac{100-\%OM}{Mineral bulk density}}$ (9)

Throughout the dataset soil pH values were measured with different extracts and SSR. Values reported as pH-KCl and pH-CaCl_2_ were transformed to pH-H_2_O values using the following equations^11,12^:

${pH}_{H2O(1:5)}=0.56+1.09\times{pH}_{KCl(1:2.5)}$ (10)

${pH}_{H2O(1:5)}=0.878+1.001\times{pH}_{CaCl2(1:5)}$ (11)

We did not correct for a difference in SSR if the pH value that had to be converted was measured at a different SSR than present in the conversion equation, as the effect is expected to be minimal due to the proton buffering capacity of the soil.

Data for P rate and N rate were divided into four categories: ≤ 25; 25-50; 50-100 and > 100 kg P ha^-1^ for P rate; and ≤ 50; 50-100; 100-200 and > 200 kg N ha^-1^ for N rate. Olsen-equivalent P values were separated into four categories: ≤ 5; 5-10; 10-25 and > 25 mg P kg^-1^. Across the world, countries use widely different Olsen P values to delimit the various agronomic P statuses. Even within a continent, the variation can be considerable: in France a value of 20 mg kg^-1^ is classified as ‘low’, whereas in Italy it is considered ‘excessive’^3^. In Australia, optimal Olsen P levels for a legume-based pasture are considered to be ~12-15 mg P kg^-113^ and critical values of between 9 and 15 mg P kg^-1^ have been established for a range of grass species^14^.In China, critical yield response values have also shown to be around this range^15^. Additionally, critical values of 23-25 mg P kg^-1^ have been reported for grass^16^. Partly to ensure there would be enough observations per category, we chose to divide the Olsen-equivalent P values into four categories, covering the range of these values (≤ 5; 5-10; 10-25 and > 25 mg P kg^-1^). To observe the interaction between P rate and soil P status, both controlling factors were divided into two categories (‘low’ ≤ 50 kg P ha^-1^ and ‘high’ > 50 kg P ha^-1^ for P rate and ‘low’ ≤ 10 mg P kg^-1^ and ‘high’ > 10 mg P kg^-1^ for SPT). A distinction was made between tropical and subtropical (latitude ≤ 35°) and temperate (latitude > 35°) climates. For soil OM content (≤ 2; 2-5; 5-10 and > 10%) and pH (≤ 5; 5-6; 6-7 and >7), four categories were also distinguished.

**Meta-analysis.** The effect size chosen to assess the response of grass and grass/legume yield to P fertilisation was the natural logarithm of the response ratio (R)^17^:

$\ln\left( r \right)=\ln\left( \frac{\bar{x_{e}}}{\bar{x_{c}}} \right)$ (12)

with $\bar{x_{e}}$ and $\bar{x_{c}}$ the means of the experimental (P fertilised) and control treatments respectively.

We also analysed the phosphorus agronomic efficiency (PAE) for each study in our dataset. This metric represents the yield increase per kg of P fertiliser and is defined as:

$PAE=\frac{\bar{x_{e}}-\bar{x_{c}}}{P rate}$ We performed a mixed‐effects meta‐analysis in r, using the rma.uni function in the “metafor” package^18^, and weighting ln(*r*) and PAE by the inverse of their variance. We estimated missing variances using the average coefficient of variation across the dataset. To ease interpretation, the results from all analyses on ln(*r*) were back‐transformed and reported as the percentage change under P fertilisation ((*r* − 1) × 100%). Both effect sizes were analysed using a mixed-effects model to calculate 95% confidence intervals^19^. For all analyses, we inferred an effect of P fertilisation if the 95% CI of the mean effect size did not overlap with 0. We used a Wald test to determine whether treatment effects were statistically different between study categories. To test for possible interactions among controlling factors, Spearman’s rank correlation coefficient was calculated for the relationships between factors.

Rosenthal’s Fail-safe N technique at *p* = 0.05 was used to test the potential impact of publication bias^20^. The fail-safe number showed that around 1,650,000 studies with null-results (i.e. with an effect size of 0) would be needed to make the mean yield response non-significant. This is remarkably higher than the critical value (5n + 10 = 6145, with n as the number of side-by-side comparisons included in the meta-analysis) suggested by Rosenthal. Therefore, it is unlikely that publication bias existed in the literature to a sufficient extent to affect the overall statistical significance of our results.

**Limitations of our study**

This study presents an overview of the efficiency of P fertiliser applications on grassland production across the world and explores the underlying factors and soil properties. Although grasslands from all continents (except Antarctica) were incorporated, there was a strong underrepresentation of African studies; only one study from Nigeria could be included. Considering the large expected population growth for this continent, future research should focus on phosphorus use on African grasslands.

Besides the factors shown in Table 1, other factors that were not taken into account in this analysis may determine the success of P fertilisation of grasslands. Globally, a total amount of 45.9 Mt P_2_O_5_ is applied as mineral fertiliser P on the world’s agricultural land^21^. Of this total, 1.8 Mt P_2_O_5_ is applied to grasslands, which amounts to 4%. Most of this fertiliser is applied in China (610 kt), Australia (320 kt), New Zealand (319 kt), and the European Union (259 kt)^21^. These numbers are small compared to the amount of P that is applied as manure, which has been estimated to be up to 20 times as high globally in intensive grassland/livestock systems^22^. In our meta-analysis there were only three studies that used organic fertiliser as source of P. Although we found more published research on manure (or other forms of organic fertiliser) using the search query described earlier, these studies usually did not account and correct for the other nutrients applied alongside P, and could therefore not be included in this meta-analysis. Depending on the origin, treatment, and composition of manure sources, the response of grass to manure applications may differ from that to mineral fertilisers such as triple superphosphate^23,24^. Animal manure sources usually contain a variety of organic and inorganic P forms that have to be mineralised and dissolve before P is available for plant uptake^25^. Besides the form in which P is applied, the timing and method of application of P fertiliser can greatly influence the crop response. We had no consistent information on either application method or timing, so these factors were not included in the meta-analysis.

Many of the controlling factors that were examined in our meta-analysis are soil properties or other environmental factors that are often closely related. Disregarding these relationships can lead to misinterpretation of results as some factors may act as surrogates for others. In our dataset, latitude and mean annual temperature were strongly correlated (ρ = -0.95; Supplementary Figure 4), as is to be expected. Grasslands in warmer areas also generally had lower amounts of OM, which may be explained by higher mineralisation rates, and lower SPT. This corresponds with the analysis of these factors individually, as shown in Figures 2 and 3. There was a relatively strong correlation between SPT and soil OM content (ρ = 0.71), whereas responses to P application were strongest under soils with low SPT and high OM content. This indicates that these factors have separate effects and do not act as surrogates. The correlation between SPT and pH or clay content was less prominent. Nevertheless, the results presented in this meta-analysis should be interpreted with these relationships in mind.

Furthermore, there are other soil factors that influence the availability of P to plants, and are thus likely to affect the success of P fertilisation. Most predominantly, the quantities of Al and Fe oxides in soil, which can act as an adsorbent for P, often strongly governs the amount of P that is available to crops. Generally, there was a lack of information on these quantities: from the studies in our database, as merely 11 studies reported some form of information on the soil content of Al and Fe oxides. Climate can also play an important role, for example by leading to water and/or heat stress, which is why parameters like soil temperature and soil moisture can influence the effectivity of P fertilisation. Unfortunately, these were not reported frequently enough to be included in this study. Lastly, although we considered differences between grass and grass/legume systems in this study, we did distinguish between different species, or mixtures of grass species. As grass and legume species vary in their P requirement and root uptake characteristics, so may their response to P fertilisation^26–28^.

**Supplementary Tables and Figures**

**Supplementary Table 1.** List of soil phosphorus tests and corresponding extraction procedures, as well as the conversion formulas used to compare over different studies.

| **Soil phosphorus test** | **Extractants** | **Soil : solution ratio** | **Extraction time** | **Method reference** | **Conversion formula** | **Conversion reference** |
| --- | --- | --- | --- | --- | --- | --- |
| Olsen | 0.5 M NaHCO_3_; pH 8.5 | 1:20 (w:v) | 30 min | Olsen et al. 1954^29^ | - | - |
| Ammonium lactate (P-AL) | 0.1 M ammonium lactate + 0.4 N acetic acid; pH 3.75 | 1:20 (w:v) | 2 h | Egner et al. 1960^30^ | $P_{OlsenEq}={(12.678+0.599\sqrt{P_{AL}}+0.232\sqrt{clay\%}-1.985\times pH)}^{2}$ | Otabbong et al. 2009^5^ |
| Bray P1 | 0.03 M NH_4_F + 0.025 M HCl | 1:10 (w:v) | 5 min | Bray and Kurtz 1945^31^ | $P_{OlsenEq}=0.302\times P_{BrayP1}+2.915$ | Wolf and Baker 1985^6^ |
| CO_2_-H_2_O | CO_2_-saturated water | 1:2.5 (w:v) | 1 h | Dirks and Scheffer 1930 | N/A | N/A |
| Colwell | 0.5 M NaHCO_3_; pH 8.5 | 1:100 (w:v) | 16 h | Colwell 1963^32^ | $P_{OlsenEq}=0.349\times P_{Colwell}+1.021$ | Moody et al. 2013^7^ |
| Modified Kelowna | 0.025 M acetic acid + 0.25 M ammonium acetate + 0.015 M NH_4_F; pH 4.9 | 1:10 (w:v) | 5 min | Qian et al. 1994^8^ | $P_{OlsenEq}=1.10\times P_{ModKelowna}-0.90$ | Qian et al. 1994^8^ |
| Mehlich 1 | 0.025 M H_2_SO_4_ + 0.05 M HCl; pH 1.2 | 1:5 (v:v) | 5 min | Mehlich 1953^33^ | $P_{OlsenEq}=0.658\times P_{Mehlich1}-0.408$ | Wolf and Baker 1985^6^ |
| Mehlich 3 | 0.015 M NH_4_F + 0.2 M acetic acid + 0.25 M NH_4_NO_3_ + 0.013 M HNO_3_; pH 2.5 | 1:10 (v:v) | 2 h | Mehlich 1984^34^ | $P_{OlsenEq}=0.325\times P_{Mehlich3}+2.010$ | Wolf and Baker 1985^6^ |
| Morgan | 10% sodium acetate; pH 4.8 | 1:5 (v:v) | 15 min | Morgan 1941^35^ | $P_{OlsenEq}=7.14\times P_{Morgan}-7.86$ (≤ 10 mg l^-1^)  $P_{Morgan}=5.5-0.22\times P_{OlsenEq}+0.008\times{P_{OlsenEq}}^{2}$ (> 10 mg l^-1^) | Humphreys et al. 1998^9^ |
| Modified Morgan | 10% ammonium acetate; pH 4.8 | 1:5 (v:v) | 15 min | McIntosh 1969^36^ | N/A | N/A |
| Texas A&M (NH_4_OAc-EDTA) | 1.43 M ammonium acetate + 0.025 M EDTA + 1 M HCl; pH 4.2 | 1:10 (w:v) | 30 min | Texas A&M Univ. Ext. Serv. 1980 | N/A | N/A |

**Supplementary Table 2.** List of the 67 studies included in the meta-analysis. Given for each study are the number of observations contributed, the soil phosphorus test method used, and the inclusion of the study in different subsets to test the effect of controlling factors. Full references can be found in the Supplementary References.

|  |  |  |  | **Contribution of studies to subsets^a^** | | | | | |
| --- | --- | --- | --- | --- | --- | --- | --- | --- | --- |
| **Study** | **# Obs.** |  | **Soil P Test** | **SPT** | **pH** | **OM** | | **Clay** | **N rate** |
| Alvim et al. 1992^37^ | 3 |  | Unknown |  | x | x |  | | x |
| Atul-Nayyar et al. 2008^38^ | 2 |  | Olsen | x | x | x |  | |  |
| Aydin and Uzun 2005^39^ | 8 |  | Olsen | x | x | x |  | | x |
| Balabanli et al. 2010^40^ | 6 |  | Olsen | x | x | x |  | | x |
| Belarmino et al. 2003^41^ | 9 |  | Unknown |  | x | x |  | | x |
| Butler and Muir 2006^42^ | 10 |  | Texas A&M |  | x |  |  | | x |
| Calviere and Duru 1999^43^ | 2 |  | None |  |  |  |  | | x |
| Caraballo et al. 1997^44^ | 1 |  | Unknown |  | x |  |  | | x |
| Cayley et al. 1998^45^ | 20 |  | None |  |  |  |  | |  |
| Coates 1994^46^ | 18 |  | Colwell | x |  |  |  | |  |
| Coates et al. 1990^47^ | 30 |  | Colwell | x |  |  |  | |  |
| Cop et al. 2009^48^ | 6 |  | Unknown |  | x | x |  | | x |
| Covacevich et al. 2006^49^ | 24 |  | Bray P1 | x | x | x |  | | x |
| Davidson et al. 2004^50^ | 4 |  | None |  | x |  | x | | x |
| Davison et al. 1997^51^ | 4 |  | Colwell | x |  |  |  | | x |
| Erkovan et al. 2010^52^ | 16 |  | Olsen | x | x | x |  | |  |
| Griffin et al. 2002^53^ | 8 |  | Modified Morgan |  |  |  |  | | x |
| Guretzky et al. 2010^54^ | 12 |  | Mehlich 3 | x | x | x |  | | x |
| Haby 2002^55^ | 26 |  | Texas A&M |  | x |  |  | | x |
| Haynes and Williams 1992^56^ | 2 |  | None |  |  |  |  | |  |
| Hejcman et al. 2012^57^ | 8 |  | Mehlich 3 | x | x |  |  | | x |
| Hendricksen et al. 1994^58^ | 2 |  | Colwell | x |  |  |  | |  |
| Hillard et al. 1992^59^ | 54 |  | Texas A&M |  | x |  |  | | x |
| Honsova et al. 2007^60^ | 41 |  | None |  | x |  |  | |  |
| Jacobsen and Surber 1995^61^ | 6 |  | Olsen | x |  |  |  | | x |
| Jones and Betteridge 1994^62^ | 2 |  | Unknown |  |  |  |  | |  |
| Jones et al. 1995^63^ | 96 |  | Olsen | x | x | x | x | |  |
| Junquan et al. 2007^64^ | 3 |  | Bray P1 | x | x |  |  | |  |
| Kering et al. 2012^65^ | 6 |  | Unknown |  | x | x |  | | x |
| Kirkham and Wilkins 1994^66^ | 4 |  | None |  | x |  |  | | x |
| Koc 2013^67^ | 8 |  | Olsen | x | x | x |  | |  |
| Liebisch et al. 2013^68^ | 4 |  | CO_2_-H_2_O |  | x | x | x | | x |
| Liebisch et al. 2014^69^ | 1 |  | None |  |  |  | x | | x |
| Lkhagvasuren et al. 2011^70^ | 24 |  | Modified Kelowna | x | x | x |  | | x |
| Loeppky et al. 1999^71^ | 8 |  | Olsen | x |  |  |  | | x |
| Martiniello and Berardo 2007^72^ | 5 |  | Olsen | x | x | x | x | | x |
| Massey et al. 2015^73^ | 3 |  | Morgan | x |  | x | x | | x |
| Mendoza et al. 2016^74^ | 2 |  | Bray P1 | x | x | x | x | |  |
| Messiga et al. 2014^75^ | 24 |  | Mehlich 3 | x |  |  |  | | x |
| Michael et al. 1991^76^ | 6 |  | None |  | x |  |  | | x |
| Michalk et al. 1998^77^ | 12 |  | None |  |  |  |  | |  |
| Morton et al. 1998^78^ | 10 |  | Olsen | x | x |  |  | |  |
| Moyer et al. 1995^79^ | 2 |  | Bray P1 | x | x |  |  | | x |
| Mullen et al. 2000^80^ | 20 |  | Mehlich 3 | x | x |  |  | |  |
| Obour et al. 2011^81^ | 6 |  | Mehlich 1 | x | x |  |  | | x |
| Paynter and Dampney 1991^82^ | 2 |  | Olsen | x | x |  |  | | x |
| Peters et al. 1997^83^ | 24 |  | Bray P1 | x | x | x | x | |  |
| Polat et al. 2007^84^ | 15 |  | Unknown |  | x | x |  | | x |
| Potthast et al. 2012^85^ | 2 |  | Bray P1 | x |  |  |  | | x |
| Power et al. 2005^86^ | 120 |  | Olsen | x | x |  |  | | x |
| Qi et al. 2015^87^ | 2 |  | Olsen | x | x | x |  | |  |
| Rao et al. 1996^88^ | 80 |  | Olsen | x | x | x | x | | x |
| Rasmussen 1995^89^ | 1 |  | None |  |  | x | x | | x |
| Romero and Marquez 2002^90^ | 3 |  | None |  | x | x |  | |  |
| Saarela et al. 2006^91^ | 22 |  | Olsen | x | x | x | x | | x |
| Sale et al. 1997^92^ | 204 |  | Colwell | x | x | x |  | |  |
| Schellberg et al. 1999^93^ | 6 |  | None |  | x |  |  | | x |
| Schils and Snijders 2004^94^ | 15 |  | P-AL | x | x | x |  | | x |
| Scholefield et al. 1999^95^ | 20 |  | None |  | x |  |  | |  |
| Seither et al. 2014^96^ | 4 |  | None |  | x |  |  | | x |
| Sheil et al. 2016^97^ | 96 |  | Morgan | x | x | x | x | | x |
| Shi et al. 2016^98^ | 4 |  | Olsen | x | x | x |  | | x |
| Smith et al. 2012^99^ | 6 |  | None |  |  |  |  | |  |
| Stroia et al. 2007^100^ | 2 |  | Olsen | x | x | x | x | | x |
| Sweeney et al. 1996^101^ | 2 |  | Bray P1 | x | x | x |  | | x |
| Wedderburn et al. 2005^102^ | 2 |  | Olsen | x | x |  |  | |  |
| Yolcu et al. 2010^103^ | 27 |  | Unknown |  | x | x |  | | x |

^a^ An ‘x’ indicates that a study was incorporated in a subset to test the corresponding controlling factor.

**Supplementary Table 3.** Results of a meta-analysis on the effect of P fertilisation on plant biomass. Results are shown for categories based on P rate, Climate, Continent, P status, Soil pH, Soil OM content, Soil Clay content, and N rate, including the nature of the effect, the size of the effect, the p-value, significant differences between categories, and the confidence interval.

| **Controlling factors** | **Categories** | **Studies and observations** | | **Effect^a^** | | | **p-value** | **CI-min^b^** | **CI-max** |
| --- | --- | --- | --- | --- | --- | --- | --- | --- | --- |
| **Overall average** |  | | **(67/1227)** | **+** | **36.7%** |  | **< 0.001** | **33.3%** | **40.2%** |
| **Crop** | Grass | | (37/696) | + | 25.1% | a | < 0.001 | 21.1% | 29.2% |
|  | Grass/legume | | (37/531) | + | 54.3% | b |  | 48.6% | 60.3% |
| **P rate** | ≤ 25 kg P ha^-1^ | | (46/523) | + | 40.4% | b | < 0.001 | 35.1% | 45.9% |
|  | 25-50 kg P ha^-1^ | | (46/500) | + | 25.7% | a |  | 21.0% | 30.7% |
|  | 50-100 kg P ha^-1^ | | (21/133) | + | 52.4% | bc |  | 41.4% | 64.3% |
|  | > 100 kg P ha^-1^ | | (7/71) | + | 65.1% | c |  | 49.0% | 82.9% |
| **Climate** | Tropical (≤ 35°) | | (22/389) | + | 45.6% | b | 0.002 | 38.9% | 52.7% |
|  | Temperate (> 35°) | | (46/838) | + | 33.3% | a |  | 29.4% | 37.3% |
| **MAT^c^** | < 10 °C | | (25/348) | + | 10.7% | a | < 0.001 | 5.9% | 15.7% |
|  | 10-20 °C | | (30/648) | + | 45.9% | b |  | 41.2% | 50.8% |
|  | > 20 °C | | (14/231) | + | 58.1% | c |  | 49.3% | 67.4% |
| **Continent** | Africa | | (1/24) | + | 80.0% | cd | < 0.001 | 54.2% | 110.1% |
|  | Asia | | (10/177) | + | 16.0% | a |  | 9.4% | 23.1% |
|  | Europe | | (19/367) | + | 14.7% | a |  | 10.4% | 19.2% |
|  | North America | | (20/327) | + | 29.1% | b |  | 23.6% | 34.8% |
|  | Oceania | | (11/300) | + | 94.8% | d |  | 85.9% | 104.1% |
|  | South America | | (8/48) | + | 58.2% | c |  | 41.9% | 76.4% |
| **P status** | ≤ 5 mg P kg^-1^ | | (16/269) | + | 109.7% | c | < 0.001 | 99.4% | 120.4% |
|  | 5-10 mg P kg^-1^ | | (14/242) | + | 15.6% | ab |  | 9.9% | 21.7% |
|  | 10-25 mg P kg^-1^ | | (10/245) | + | 25.3% | b |  | 19.2% | 31.6% |
|  | > 25 mg P kg^-1^ | | (5/168) | + | 6.5% | a |  | 0.3% | 13.2% |
| **P status × P rate^d^** | Low SPT; low P rate | | (25/457) | + | 50.3% | b | < 0.001 | 44.1% | 56.8% |
|  | Low SPT; high P rate | | (7/54) | + | 129.1% | c |  | 102.8% | 158.8% |
|  | High SPT; low P rate | | (14/365) | + | 14.5% | a |  | 9.4% | 19.8% |
|  | High SPT; high P rate | | (3/48) | + | 49.2% | b |  | 30.9% | 69.9% |
| **Soil pH** | ≤ 5 | | (5/92) | + | 25.7% | b | < 0.001 | 17.4% | 34.7% |
|  | 5-6 | | (24/478) | + | 60.0% | c |  | 54.9% | 65.2% |
|  | 6-7 | | (15/280) | + | 16.4% | ab |  | 11.9% | 21.2% |
|  | > 7 | | (14/226) | + | 10.7% | a |  | 5.6% | 15.9% |
| **Soil OM content** | ≤ 2 % | | (7/132) | + | 10.9% | a | < 0.001 | 3.0% | 19.4% |
|  | 2-5 % | | (18/230) | + | 40.9% | b |  | 33.3% | 49.0% |
|  | 5-10 % | | (8/353) | + | 56.3% | c |  | 49.6% | 63.4% |
|  | >10 % | | (3/16) | + | 80.2% | c |  | 46.5% | 121.6% |
| **Soil clay content** | ≤ 10 % | | (3/89) | 0 | 6.6% | a | < 0.001 | -1.1% | 14.7% |
|  | 10-25 % | | (9/202) | + | 37.9% | b |  | 31.6% | 44.5% |
|  | > 25 % | | (5/33) | + | 75.3% | c |  | 55.5% | 97.7% |
| **N rate** | ≤ 50 kg N ha^-1^ | | (19/127) | + | 19.8% | b | < 0.001 | 14.8% | 25.0% |
|  | 50-100 kg N ha^-1^ | | (21/169) | + | 20.6% | b |  | 16.7% | 24.6% |
|  | 100-200 kg N ha^-1^ | | (15/96) | + | 18.2% | b |  | 12.9% | 23.7% |
|  | > 200 kg N ha^-1^ | | (11/275) | + | 11.2% | a |  | 8.3% | 14.1% |

^a^ For each of the weighting functions and subgroups a + indicates a significant positive effect (95% confidence interval > 0) and a 0 indicates non-significance (95% confidence interval overlaps 0). Letters indicate significant differences within categories. Values with the same letter per controlling factor, per weighting method, are not significantly different.

^b^ CI-min indicates the lower limit of the 95% confidence interval; CI-max indicates the upper limit of the 95% confidence interval.

^c^ MAT = mean annual temperature

^d^ Low SPT: Olsen-equivalent P status ≤ 10 mg P kg^-1^; High SPT: Olsen-equivalent P status > 10 mg P kg^-1^; low P rate: ≤ 50 kg P ha^-1^; high P rate: > 50 kg P ha^-1^.

**Supplementary Table 4.** Results of a meta-analysis on the effect of P fertilisation on P agronomic efficiency. Results are shown for categories based on P rate, Climate, Continent, P status, Soil pH, Soil OM content, Soil Clay content, and N rate, including the nature of the effect, the size of the effect, the p-value, significant differences between categories, and the confidence interval.

| **Controlling factors** | **Categories** | **Studies and observations** | | **PAE^a^** | | | **p-value** | **CI-min^b^** | **CI-max** |
| --- | --- | --- | --- | --- | --- | --- | --- | --- | --- |
| **Overall average** |  | | **(67/1227)** | **+** | **32.0** |  | **< 0.001** | **29.5** | **34.4** |
| **Crop** | Grass | | (37/696) | + | 22.2 | a | < 0.001 | 19.2 | 25.2 |
|  | Grass/legume | | (37/531) | + | 46.3 | b |  | 42.5 | 50.1 |
| **P rate** | ≤ 25 kg P ha^-1^ | | (46/523) | + | 52.8 | c | < 0.001 | 48.1 | 57.5 |
|  | 25-50 kg P ha^-1^ | | (46/500) | + | 24.8 | b |  | 21.4 | 28.2 |
|  | 50-100 kg P ha^-1^ | | (21/133) | + | 30.8 | b |  | 24.8 | 36.7 |
|  | > 100 kg P ha^-1^ | | (7/71) | + | 11.9 | a |  | 4.8 | 19.0 |
| **Climate** | Tropical (≤ 35°) | | (22/389) | + | 33.8 |  | 0.31 | 29.6 | 38.0 |
|  | Temperate (> 35°) | | (46/838) | + | 31.1 |  |  | 28.1 | 34.2 |
| **Continent** | Africa | | (1/24) | + | 117.2 | e | < 0.001 | 91.1 | 137.3 |
|  | Asia | | (10/177) | + | 11.9 | a |  | 7.0 | 16.7 |
|  | Europe | | (19/367) | + | 23.3 | b |  | 19.5 | 27.1 |
|  | North America | | (20/327) | + | 17.1 | a |  | 13.3 | 21.0 |
|  | Oceania | | (11/300) | + | 74.3 | d |  | 69.0 | 79.6 |
|  | South America | | (8/48) | + | 61.0 | c |  | 49.4 | 72.5 |
| **P status** | ≤ 5 mg P kg^-1^ | | (16/269) | + | 74.7 | c | < 0.001 | 69.2 | 80.3 |
|  | 5-10 mg P kg^-1^ | | (14/242) | + | 16.3 | a |  | 11.1 | 21.5 |
|  | 10-25 mg P kg^-1^ | | (10/245) | + | 26.9 | b |  | 21.9 | 31.9 |
|  | > 25 mg P kg^-1^ | | (5/168) | + | 8.7 | a |  | 2.7 | 14.7 |
| **P status × P rate^c^** | Low SPT; low P rate | | (25/457) | + | 46.2 | b | < 0.001 | 41.5 | 50.9 |
|  | Low SPT; high P rate | | (7/54) | + | 50.5 | b |  | 40.1 | 60.8 |
|  | High SPT; low P rate | | (14/365) | + | 22.3 | a |  | 17.5 | 27.1 |
|  | High SPT; high P rate | | (3/48) | + | 10.9 | a |  | 0.9 | 20.9 |
| **Soil pH** | ≤ 5 | | (5/92) | + | 19.7 | b | < 0.001 | 12.6 | 26.9 |
|  | 5-6 | | (24/478) | + | 49.9 | c |  | 46.2 | 53.6 |
|  | 6-7 | | (15/280) | + | 24.2 | b |  | 19.6 | 28.8 |
|  | > 7 | | (14/226) | + | 9.7 | a |  | 5.2 | 14.2 |
| **Soil OM content** | ≤ 2 % | | (7/132) | 0 | 7.2 | a | < 0.001 | -0.1 | 14.4 |
|  | 2-5 % | | (18/230) | + | 41.5 | b |  | 35.4 | 47.7 |
|  | 5-10 % | | (8/353) | + | 59.2 | c |  | 53.9 | 64.5 |
|  | >10 % | | (3/16) | + | 64.1 | c |  | 44.8 | 83.4 |
| **Soil clay content** | ≤ 10 % | | (3/89) | 0 | 3.3 | a | < 0.001 | -1.3 | 7.9 |
|  | 10-25 % | | (9/202) | + | 32.8 | b |  | 28.7 | 37.0 |
|  | > 25 % | | (5/33) | + | 31.5 | b |  | 23.3 | 39.8 |
| **N rate** | ≤ 50 kg N ha^-1^ | | (19/127) | + | 12.8 |  | 0.13 | 8.5 | 17.1 |
|  | 50-100 kg N ha^-1^ | | (21/169) | + | 15.8 |  |  | 12.0 | 19.6 |
|  | 100-200 kg N ha^-1^ | | (15/96) | + | 21.9 |  |  | 15.0 | 28.8 |
|  | > 200 kg N ha^-1^ | | (11/275) | + | 17.8 |  |  | 13.8 | 21.8 |

^a^ For each of the subgroups a + indicates a significant positive effect (95% confidence interval > 0) and a 0 indicates non-significance (95% confidence interval overlaps 0). Letters indicate significant differences within categories. Values with the same letter per controlling factor, per weighting method, are not significantly different.

^b^ CI-min indicates the lower limit of the 95% confidence interval; CI-max indicates the upper limit of the 95% confidence interval.

^c^ Low SPT: Olsen-equivalent P status ≤ 10 mg P kg^-1^; High SPT: Olsen-equivalent P status > 10 mg P kg^-1^; low P rate: ≤ 50 kg P ha^-1^; high P rate: > 50 kg P ha^-1^.


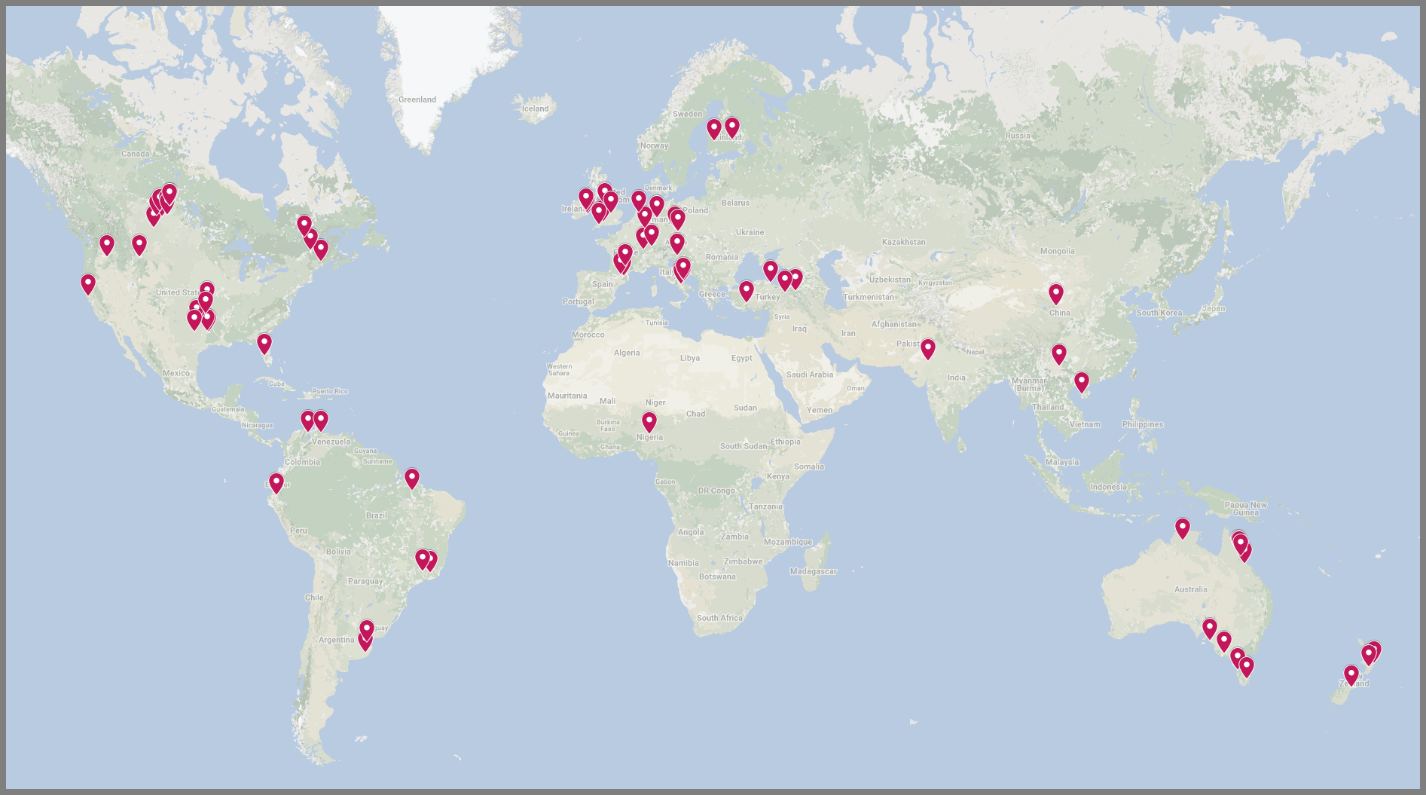


**Supplementary Figure 1.** Map with the distribution and approximate locations of the 77 experimental sites used in the 67 studies incorporated in this meta-analysis. Note that several studies had more than one study site, and that some locations were used by multiple studies. The image was generated using Google Maps, and is available in more detail (https://www.google.com/maps/d/viewer?mid=1lAkTLvnkB-Gb7HD4NufYFo36-Ak&usp=sharing).

**Supplementary Figure 2.** Distribution of different soil phosphorus test methods used to measure initial soil phosphorus status over the studies included in this meta-analysis. The study count is represented by the broad bars, whereas the narrow bars show the corresponding number of data points. For methods with a green bar, the data were transformed to Olsen-equivalent values and subsequently incorporated in the Soil P subset. Short summaries of the different SPT methods can be found in Supplementary Table 1.

**Supplementary Figure 3.** Soil phosphorus (P) status (as Olsen-equivalent mg P kg^-1^) over different categories of P application rate (a; 39 studies, 924 observations) and soil pH (b; 30 studies, 827 observations). The black dots represent the P status of the soils at the start of the P fertilisation experiments underlying the calculated yield effects and the red diamonds indicate the averages (for both x and y variables) per defined category. The dotted lines indicate category limits for P application rate (≤ 25; 25-50; 50-100; > 100 kg P ha^-1^) and pH (≤ 5; 5-6; 6-7; > 7).

**≤ 25 25-50 50-100 > 100 kg ha^-1^**

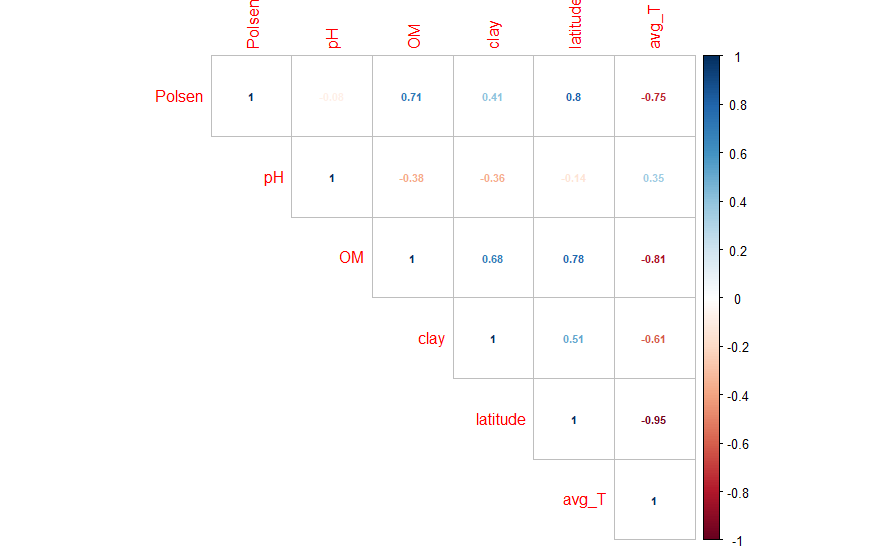


**Supplementary Figure 4.** Spearman’s rank correlation test results (ρ) for the various controlling factors used in our meta-analysis. Polsen = Olsen-equivalent SPT; avg_T = mean annual temperature. Numbers close to 1 indicate a strong positive correlation and numbers close to -1 a strong negative correlation. Numbers close to 0 indicate a lack of correlation.

**Supplementary Figure 5.** Absolute yield response (in tonnes ha^-1^) as a function of the soil phosphorus (P) status (as Olsen-equivalent mg P kg^-1^) over different categories of soil P status (39 studies, 924 observations). The black dots represent the P status of the soils at the start of the P fertilisation experiments underlying the calculated yield effects and the red diamonds indicate the averages (for both x and y variables) per defined soil P status category. The dotted lines indicate category limits for soil P status (≤ 5; 5-10; 10-25; > 25 mg P kg^-1^).

**References**

1. Climate data for cities worldwide - Climate-Data.org. Available at: https://en.climate-data.org/. (Accessed: 26th June 2020)

2. Nawara, S. *et al.* A comparison of soil tests for available phosphorus in long-term field experiments in Europe. *Eur. J. Soil Sci.* **68**, 873–885 (2017).

3. Jordan-Meille, L. *et al.* An overview of fertilizer-P recommendations in Europe: Soil testing, calibration and fertilizer recommendations. *Soil Use Manag.* **28**, 419–435 (2012).

4. Sharpley, A. *et al.* Phosphorus indices: Why we need to take stock of how we are doing. *J. Environ. Qual.* **41**, 1711–1719 (2012).

5. Otabbong, E., Börling, K., Kätterer, T. & Mattssona, L. Compatibility of the ammonium lactate (AL) and sodium bicarbonate (Olsen) methods for determining available phosphorus in Swedish soils. *Acta Agric Scand Sect B Soil Plant Sci* **59**, 373–378 (2009).

6. Wolf, A. M. & Baker, D. E. Comparisons of soil test phosphorus by Olsen, Bray P1, Mehlich I and Mehlich III methods 1. *Commun. Soil Sci. Plant Anal.* **16**, 467–484 (1985).

7. Moody, P. W., Speirs, S. D., Scott, B. J. & Mason, S. D. Soil phosphorus tests I: What soil phosphorus pools and processes do they measure? *Crop Pasture Sci.* **64**, 461–468 (2013).

8. Qian, P., Schoenaru, J. J. & Karamanos, R. E. Simultaneous extraction of available phosphorus and potassium with a new soil test: A modification of Kelowna extraction. *Commun. Soil Sci. Plant Anal.* **25**, 627–635 (1994).

9. Humphreys, J., Tunney, H. & Duggan, P. Soil phosphorus determination using three extraction procedures, the effect of sampling depth and comparison of phosphorus fertiliser recommendations for grassland. *Irish J. Agric. Food Res.* **37**, 29–38 (1998).

10. Rawls, W. J. Estimating soil bulk density from particle size analysis and organic matter content. *Soil Sci.* **135**, 123–125 (1983).

11. Ahern, C. R., Baker, D. E. & Aitken, R. L. Models for relating pH measurements in water and calcium chloride for a wide range of pH, soil types and depths. *Plant Soil* **171**, 47–52 (1995).

12. Kabala, C., Musztyfaga, E., Galka, B., Labunska, D. & Manczynska, P. Conversion of soil pH 1:2.5 KCl and 1:2.5 H2O to 1:5 H2O: Conclusions for soil management, environmental monitoring, and international soil databases. *Polish J. Environ. Stud.* **25**, 647–653 (2016).

13. Simpson, R. J., Richardson, A. E., Nichols, S. N. & Crush, J. R. Pasture plants and soil fertility management to improve the efficiency of phosphorus fertiliser use in temperate grassland systems. *Crop Pasture Sci.* **65**, 556–575 (2014).

14. Sandral, G. A. *et al.* Field benchmarking of the critical external phosphorus requirements of pasture legumes for southern Australia. *Crop Pasture Sci.* **70**, 1080–1096 (2019).

15. Bai, Z. *et al.* The critical soil P levels for crop yield, soil fertility and environmental safety in different soil types. *Plant Soil* **372**, 27–37 (2013).

16. Johnston, A. E., Poulton, P. R. & White, R. P. Plant-available soil phosphorus. Part II: The response of arable crops to Olsen P on a sandy clay loam and a silty clay loam. *Soil Use Manag.* **29**, 12–21 (2013).

17. Hedges, L. V., Gurevitch, J. & Curtis, P. S. The meta-analysis of response ratios in experimental ecology. *Ecology* **80**, 1150–1156 (1999).

18. Viechtbauer, W. Conducting meta-analyses in R with the metafor package. *J. Stat. Softw.* **36**, 1–48 (2010).

19. Hedges, L. V. & Olkin, I. *Statistical methods for meta-analysis*. (Academic Press, London, UK, 1985).

20. Rosenthal, R. & Rosnow, R. L. *Essentials of Behavioral Research: Methods and Data Analysis*. (McGraw-Hill, 1991).

21. Heffer, P., Gruère, A. & Roberts, T. *Assessment of fertilizer use by crop at the global level*. (2017).

22. Sattari, S. Z., Bouwman, A. F., Martinez Rodríguez, R., Beusen, A. H. W. & van Ittersum, M. K. Negative global phosphorus budgets challenge sustainable intensification of grasslands. *Nat. Commun.* **7**, (2016).

23. Delin, S. Fertilizer value of phosphorus in different residues. *Soil Use Manag.* **32**, 17–26 (2016).

24. Kuligowski, K., Poulsen, T. G., Rubæk, G. H. & Sørensen, P. Plant-availability to barley of phosphorus in ash from thermally treated animal manure in comparison to other manure based materials and commercial fertilizer. *Eur. J. Agron.* **33**, 293–303 (2010).

25. Barnett, G. M. Phosphorus forms in animal manure. *Bioresour. Technol.* **49**, 139–147 (1994).

26. Hamel, S. C. & Heckman, J. R. Predicting need for phosphorus fertilizer by soil testing during seeding of cool season grasses. *HortScience* **41**, 1690–1697 (2006).

27. Paredes, C., Menezes-Blackburn, D., Cartes, P., Gianfreda, L. & Luz Mora, M. Phosphorus and nitrogen fertilization effect on phosphorus uptake and phosphatase activity in ryegrass and tall fescue grown in a Chilean andisol. *Soil Sci.* **176**, 245–251 (2011).

28. Ros, M. B. H., De Deyn, G. B., Koopmans, G. F., Oenema, O. & van Groenigen, J. W. What root traits determine grass resistance to phosphorus deficiency in production grassland? *J. Plant Nutr. Soil Sci.* **181**, 323–335 (2018).

29. Olsen, S. R., Cole, C. V & Watanabe, F. S. *Estimation of available phosphorus in soils by extraction with sodium bicarbonate*. (USDA, 1954).

30. Egnér, H., Riehm, H. & Domingo, W. R. Untersuchungen über die chemische Bodenanalyse als Grundlage für die Beurteilung des Nährstoffzustandes der Böden. II. Chemische Extraktionsmethoden zur Phosphor-und Kaliumbestimmung. *K. Lantbrukshögskolans Ann.* **26**, 199–215 (1960).

31. Bray, R. H. & Kurtz, L. T. Determination of total, organic, and available forms of phosphorus in soils. *Soil Sci.* **59**, 39–46 (1945).

32. Colwell, J. D. The estimation of the phosphorus fertilizer requirements of wheat in southern New South Wales by soil analysis. *Aust. J. Exp. Agric.* **3**, 190–198 (1963).

33. Mehlich, A. *Determination of P, Ca, Mg, K, Na, NH4*. (1953).

34. Mehlich, A. Mehlich 3 soil test extractant: A modification of Mehlich 2 extractant. *Commun. Soil Sci. Plant Anal.* **15**, 1409–1416 (1984).

35. Morgan, M. F. *Chemical soil diagnosis by the universal soil testing system*. (University of Connecticut, 1941).

36. McIntosh, J. L. Bray and Morgan Soil Extractants Modified for Testing Acid Soils from Different Parent Materials1. *Agron. J.* **61**, 259–265 (1969).

37. Alvim, M. J., Carvalho, M. M. & Botrel, M. de A. NPK fertilization to establish setaria on a lowland soil (in Portuguese). *Pesqui. Agropecuária Bras.* **27**, 79–85 (1992).

38. Atul-Nayyar *et al.* Arbuscular mycorrhizal fungi and nematodes are involved in negative feedback on a dual culture of alfalfa and Russian wildrye. *Appl. Soil Ecol.* **40**, 30–36 (2008).

39. Aydin, I. & Uzun, F. Nitrogen and phosphorus fertilization of rangelands affects yield, forage quality and the botanical composition. *Eur. J. Agron.* **23**, 8–14 (2005).

40. Balabanli, C., Albayrak, S. & Yuksel, O. Effects of nitrogen, phosphorus and potassium fertilization on the quality and yield of native rangeland. *Turkish J. F. Crop.* **15**, 164–168 (2010).

41. Belarmino, M. C. J., Pinto, J. C., Rocha, G. P., Furtini Neto, A. E. & De Morais, A. R. Tanzania grass tiller height and dry matter production under different doses of simple superphosphate and ammonium sulfate (in Portuguese). *Ciência e Agrotecnologia* **27**, 879–885 (2003).

42. Butler, T. J. & Muir, J. P. Dairy manure compost improves soil and increases tall wheatgrass yield. *Agron. J.* **98**, 1090–1096 (2006).

43. Calvière, I. & Duru, M. The effect of N and P fertilizer application and botanical composition on the leaf/stem ratio patterns in spring in Pyrenean meadows. *Grass Forage Sci.* **54**, 255–266 (1999).

44. Caraballo, A., Morillo, D. E., Faría‐Mármol, J. & McDowell, L. R. Frequency of defoliation and nitrogen and phosphorus fertilization on Andropogon gayanus Kunth. I. Yield, crude protein content, and in vitro digestibility. *Commun. Soil Sci. Plant Anal.* **28**, 823–831 (1997).

45. Cayley, J. W. D., Hannah, M. C., Kearney, G. A. & Clark, S. G. Effects of phosphorus fertiliser and rate of stocking on the seasonal pasture production of perennial tyegrass-subterranean clover pasture. *Aust. J. Agric. Res.* **49**, 233–248 (1998).

46. Coates, D. B. The effect of phosphorus as fertiliser or supplement on pasture and cattle productivity in the semi-arid tropics of north Queensland. *Trop. Grasslands* **28**, 90–108 (1994).

47. Coates, D. B., Kerridge, P. C., Miller, C. P. & Winter, W. H. Phosphorus and beef production in northern Australia. 7. The effect of phosphorus on the composition, yield and quality of legume-based pasture and their relation to animal production. *Trop. Grasslands* **24**, 209–220 (1990).

48. Čop, J., Vidrih, M. & Hacin, J. Influence of cutting regime and fertilizer application on the botanical composition, yield and nutritive value of herbage of wet grasslands in Central Europe. *Grass Forage Sci.* **64**, 454–465 (2009).

49. Covacevich, F., Marino, M. A. & Echeverría, H. E. The phosphorus source determines the arbuscular mycorrhizal potential and the native mycorrhizal colonization of tall fescue and wheatgrass. *Eur. J. Soil Biol.* **42**, 127–138 (2006).

50. Davidson, E. A. *et al.* Nitrogen and phosphorus limitation of biomass growth in a tropical secondary forest. *Ecol. Appl.* **14**, 150–163 (2004).

51. Davison, T. M., Orr, W. N., Silver, B. A., Walker, R. G. & Duncalfe, F. Phosphorus fertilizer for nitrogen fertilized dairy pastures. 1. Long term effects on pasture, diet and soil. *J. Agric. Sci.* **129**, 205–217 (1997).

52. Erkovan, H. I., Güllap, M. K., Daşçi, M. & Koç, A. Effects of phosphorus fertilizer and phosphorus solubilizing bacteria applications on clover dominant meadow: I. Hay yield and botanical composition. *Turkish J. F. Crop.* **15**, 12–17 (2010).

53. Griffin, R. A. & Jurinak, J. J. Estimation of activity coefficients from the electrical conductivity of natural aquatic systems and soil extracts. *Soil Sci.* **116**, 26–30 (1973).

54. Guretzky, J., Kering, M., Mosali, J., Funderburg, E. & Biermacher, J. T. Fertilizer rate effects on forage yield stability and nutrient uptake of midland Bermudagrass. *J. Plant Nutr.* **33**, 1819–1834 (2010).

55. Haby, V. A. Soil fertility and management of acid coastal plain soils for crop production. *Commun. Soil Sci. Plant Anal.* **33**, 2497–2520 (2002).

56. Haynes, R. J. & Williams, P. H. Long-term effect of superphosphate on accumulation of soil phosphorus and exchangeable cations on a grazed, irrigated pasture site. *Plant Soil* **142**, 123–133 (1992).

57. Hejcman, M., Strnad, L., Hejcmanova, P. & Pavlů, V. Response of plant species composition, biomass production and biomass chemical properties to high N, P and K application rates in Dactylis glomerata- and Festuca arundinacea-dominated grassland. *Grass Forage Sci.* **67**, 488–506 (2012).

58. Hendricksen, R. E., Ternouth, J. H. & Punter, L. D. Seasonal nutrient intake and phosphorus kinetics of grazing steers in northern Australia. *Aust. J. Agric. Res.* **45**, 1817–1829 (1994).

59. Hillard, J. B., Haby, V. A. & Hons, F. M. Annual ryegrass response to limestone and phosphorus on an Ultisol. *J. Plant Nutr.* **15**, 1253–1268 (1992).

60. Honsová, D. *et al.* Species composition of an alluvial meadow after 40 years of applying nitrogen, phospohorus and potassium fertilizer. *Preslia* **79**, 245–258 (2007).

61. Jacobsen, J. S. & Surber, G. W. Alfalfa/grass response to nitrogen and phosphorus applications. *Commun. Soil Sci. Plant Anal.* **26**, 1273–1282 (1995).

62. Jones, R. J. & Betteridge, K. Effect of superphosphate, or its component elements (phosphorus, sulfur, and calcium), on the grazing preference of steers on a tropical grass-legume pasture grown on a low phosphorus soil. *Aust. J. Exp. Agric.* **34**, 349–353 (1994).

63. Jones, M. B., Vaughn, C. E. & Williams, W. A. Soil phosphorus requirements for maximum growth of northern California subclover-annual grass pastures. *Commun. Soil Sci. Plant Anal.* **26**, 197–207 (1995).

64. Junquan, Z. *et al.* Effect of phosphorus, potassium and lime application on pasture in acid soil in Yunnan Province, China. *New Zeal. J. Agric. Res.* **50**, 523–535 (2007).

65. Kering, M. K., Biermacher, J. T., Butler, T. J., Mosali, J. & Guretzky, J. A. Biomass yield and nutrient responses of switchgrass to phosphorus application. *Bioenergy Res.* **5**, 71–78 (2012).

66. Kirkham, F. W. & Wilkins, R. J. The productivity and response to inorganic fertilizers of species-rich wetland hay meadows on the Somerset Moors: the effect of nitrogen, phosphorus and potassium on herbage production. *Grass Forage Sci.* **49**, 163–175 (1994).

67. Koc, A. Effect of phosphorus doses and application time on the yield and quality of hay and botanical composition of clover dominant meadow in highlands of Turkey. *Turkish J. F. Crop.* **18**, 205–210 (2013).

68. Liebisch, F. *et al.* Plant phosphorus nutrition indicators evaluated in agricultural grasslands managed at different intensities. *Eur. J. Agron.* **44**, 67–77 (2013).

69. Liebisch, F. *et al.* Seasonal dynamics and turnover of microbial phosphorusin a permanent grassland. *Biol. Fertil. Soils* **50**, 465–475 (2014).

70. Lkhagvasuren, B., Schoenau, J. J., Anderson, D. W. & Malhi, S. S. Plant and soil responses to nitrogen and phosphorus fertilization of bromegrass-dominated haylands in Saskatchewan, Canada. *Grass Forage Sci.* **66**, 351–360 (2011).

71. Loeppky, H. A., Horton, P. R., Bittman, S., Wright, T. & Nuttall, W. F. Forage seed yield response to N and P fertilizers and soil nutrients in northeastern Saskatchewan. *Can. J. Soil Sci.* **79**, 265–271 (1999).

72. Martiniello, P. & Berardo, N. Residual fertilizer effects on dry-matter yield and nutritive value of Mediterranean pastures. *Grass Forage Sci.* **62**, 87–99 (2007).

73. Massey, P. A., Creamer, R. E., Whelan, M. J. & Ritz, K. Insensitivity of soil biological communities to phosphorus fertilization in intensively managed grassland systems. *Grass Forage Sci.* **71**, 139–152 (2015).

74. Mendoza, R., Bailleres, M., García, I. & Ruiz, O. Phosphorus fertilization of a grass-legume mixture: Effect on plant growth, nutrients acquisition and symbiotic associations with soil microorganisms. *J. Plant Nutr.* **39**, 691–701 (2016).

75. Messiga, A. J., Ziadi, N., Bélanger, G. & Morel, C. Relationship between soil phosphorus and phosphorus budget in grass sward with varying nitrogen applications. *Soil Sci. Soc. Am. J.* **78**, 1481–1488 (2014).

76. Michael, N., Bradshaw, A. D. & Hall, J. E. The value of fertilizer, surface applied and injected sewage sludge to vegetation established on reclaimed colliery spoil suffering from regression. *Soil Use Manag.* **7**, 233–239 (1991).

77. Michalk, D. L., Nan-Ping, F. & Chin-Ming, Z. Improvement of dry tropical rangelands in Hainan Island, China: 4. Effect of seedbed on pasture establishment. *J. Range Manag.* **51**, 106–114 (1998).

78. Morton, J. D., Wheeler, D. M. & Smith, N. S. Effect of lime and form of superphosphate on productivity of dryland pastures. *New Zeal. J. Agric. Res.* **41**, 65–74 (1998).

79. Moyer, J. L., Sweeney, D. W. & Lamond, R. E. Response of tall fescue to fertilizer placement at different levels of phosphorus, potassium, and soil pH. *J. Plant Nutr.* **18**, 729–746 (1995).

80. Mullen, R. W., Phillips, S. B., Raun, W. R., Johnson, G. V. & Thomason, W. E. Forage yield and crude protein of interseeded legume-bermudagrass mixtures as affected by phosphorus fertilizer. *J. Plant Nutr.* **23**, 673–681 (2000).

81. Obour, A. K. *et al.* Agronomic and environmental impacts of phosphorus fertilization of low input bahiagrass systems in Florida. *Nutr. Cycl. Agroecosystems* **89**, 281–290 (2011).

82. Paynter, R. M. & Dampney, P. M. R. The effect of rate and timing of phosphate fertilizer on the yield and phosphate off take of grass grown for silage at moderate to high levels of soil phosphorus. *Grass Forage Sci.* **46**, 131–137 (1991).

83. Peters, M., Tarawali, S. A. & Alkämper, J. Dry season performance of four tropical pasture legumes in subhumid west Africa as influenced by superphosphate application and weed control. *Trop. Grasslands* **31**, 201–213 (1997).

84. Polat, T., Bükün, B. & Okant, M. Dose response effect of nitrogen and phosphorus on forage quality, yield and economic return of rangelands. *Pakistan J. Bot.* **39**, 807–816 (2007).

85. Potthast, K., Hamer, U. & Makeschin, F. In an Ecuadorian pasture soil the growth of Setaria sphacelata, but not of soil microorganisms, is co-limited by N and P. *Appl. Soil Ecol.* **62**, 103–114 (2012).

86. Power, V., Tunney, H. & Jeffrey, D. W. The phosphorus requirements for silage production on high fertility soils. *Irish J. Agric. Food Res.* **44**, 281–296 (2005).

87. Qi, J., Nie, Z., Jiao, T. & Zhang, D. Phosphorus and defoliation interact and improve the growth and composition of the plant community and soil properties in an alpine pasture of qinghai-tibet plateau. *PLoS One* **10**, (2015).

88. Rao, A. S., Singh, K. C. & Wight, J. R. Productivity of Cenchms ciliaris in relation to rain- fall and fertilization. *J. Range Manag.* **49**, 143–146 (1996).

89. Rasmussen, P. E. Effects of fertilizer and stubble burning on downy brome competition in winter wheat. *Commun. Soil Sci. Plant Anal.* **26**, 951–960 (1995).

90. Romero, C. & Márquez, O. Effect of phosphorus in grass Brachiaria humidicola on the milky production of cow’s double purpose (in Spanish). *Rev. Científica* **12**, 578–580 (2002).

91. Saarela, I., Huhta, H. & Virkajärvi, P. Effects of repeated phosphorus fertilisation on field crops in Finland 2. Sufficient phosphorus application rates on silty and sandy soils. *Agric. Food Sci.* **15**, 423–443 (2006).

92. Sale, P. W. G. *et al.* The agronomic effectiveness of reactive phosphate rocks 1. Effect of the pasture environment. *Aust. J. Exp. Agric.* **37**, 921–936 (1997).

93. Schellberg, J., Möseler, B. M., Kühbauch, W. & Rademacher, I. F. Long-term effects of fertilizer on soil nutrient concentration, yield, forage quality and floristic composition of a hay meadow in the Eifel mountains, Germany. *Grass Forage Sci.* **54**, 195–207 (1999).

94. Schils, R. & Snijders, P. The combined effect of fertiliser nitrogen and phosphorus on herbage yield and changes in soil nutrients of a grass/clover and grass-only sward. *Nutr. Cycl. Agroecosystems* **68**, 165–179 (2004).

95. Scholefield, D., Sheldrick, R. D., Martyn, T. M. & Lavender, R. H. A comparison of triple superphosphate and Gafsa ground rock phosphate fertilisers as P-sources for grass-clover swards on a poorly-drained acid clay soil. *Nutr. Cycl. Agroecosystems* **53**, 147–155 (1999).

96. Seither, M., Wrage-Mönnig, N. & Isselstein, J. Biomass production of Lolio-Cynosuretum grassland is not increased by plant-species richness. *J. Plant Nutr. Soil Sci.* **177**, 613–623 (2014).

97. Sheil, T. S. *et al.* Long-term effects of phosphorus fertilizer on soil test phosphorus, phosphorus uptake and yield of perennial ryegrass. *J. Agric. Sci.* **154**, 1068–1081 (2016).

98. Shi, Y., Ziadi, N., Hamel, C., Lajeunesse, J. & Lafond, J. Phosphorus fertilization effect on Timothy root growth, and associated arbuscular mycorrhizal development. *Agron. J.* **108**, 930–938 (2016).

99. Smith, L. C., Moss, R. A., Morton, J. D., Metherell, A. K. & Fraser, T. J. Pasture production from a long-term fertiliser trial under irrigation. *New Zeal. J. Agric. Res.* **55**, 105–117 (2012).

100. Stroia, C., Morel, C. & Jouany, C. Dynamics of diffusive soil phosphorus in two grassland experiments determined both in field and laboratory conditions. *Agric. Ecosyst. Environ.* **119**, 60–74 (2007).

101. Sweeney, D. W., Moyer, J. L. & Havlin, J. L. Multinutrient fertilization and placement to improve yield and nutrient concentration of Tall Fescue. *Agron. J.* **88**, 982–986 (1996).

102. Wedderburn, M. E., Barker, D. J., Chapman, D. F., Orr, S. J. & Dymock, N. Genetic differentiation in white clover (Trifolium repens) populations during 8 years of contrasting phosphorus supply in New Zealand hill country. *New Zeal. J. Agric. Res.* **48**, 63–74 (2005).

103. Yolcu, H., Serin, Y. & Tan, M. The effects of seeding patterns, nitrogen and phosphorus fertilizations on production and botanical composition in lucerne-smooth bromegrass mixtures. *Bulg. J. Agric. Sci.* **16**, 719–727 (2010).
